# Modern Monetary Theory: A Solid Theoretical Foundation of Economic Policy?

**DOI:** 10.1007/s11293-021-09713-6

**Published:** 2021-05-25

**Authors:** Aloys L. Prinz, Hanno Beck

**Affiliations:** 1 Institute of Public Economics, University of Muenster, Wilmergasse 6-8, 48143 Muenster, Germany; 2 University of Applied Sciences Pforzheim, Tiefenbronner Straße 65, 75175 Pforzheim, Germany

**Keywords:** Modern Monetary Theory, Keynesian cross, Public budget deficits, Fiscal policy, Monetary policy, B59, E42, E52, E62, H63

## Abstract

This paper shows that so-called modern monetary theory (MMT) lacks a sound economic foundation for its far-reaching policy recommendations. This paper’s main contribution to the literature concerns the theoretical foundation of MMT. A simple macroeconomic model shows that MMT is indistinguishable from the Keynesian cross model, as well as a neoclassical macroeconomic model, even when taking account of money in the sense of MMT. This result is in stark contrast to the claims of MMT proponents. Accordingly, it is asserted that MMT is a fundamentally new theory of money and monetary economics. However, MMT is admittedly based on the functional finance concept of the 1940s and money is modelled as an accounting identity. In addition, the fundamental connection between government expenditures for goods and services and the steady state equilibrium value of the national income, the so-called fiscal stance, is a well-known result that is not only consistent with MMT. The interpretation of the fiscal stance, in combination with the accounting identity for money, is a major issue because an equilibrium condition should have a certain causal direction of effects. Based on this reading of the equilibrium condition, policy recommendations encompass the fiscal dominance of monetary policy via monetization of public debt, a job guarantee by the state, along with a so-called Green New Deal. According to the results of this paper, these policy recommendations cannot be justified with MMT.

**Supplementary Information:**

The online version contains supplementary material available at 10.1007/s11293-021-09713-6.

## Introduction

Recently, a macroeconomic theory, modern monetary theory (MMT) (also dubbed modern money theory), has become a hot topic in United States (U.S.) politics. Stephanie Kelton, a proponent of this theory, was among the advisers of Bernie Sanders in the 2016 U.S. presidential campaign. Her contemporary, Alexandria Ocasio-Cortez, a popular member of the Democratic Party in the U.S., seems to also adhere to MMT. MMT offers politicians what they want most: a simple justification for policies they want to carry out. A case in point is active U.S. labor market policy. The U.S. public expenditures in this policy area are very low in comparison to all other countries of the Organisation for Economic Co-operation and Development (OECD) (Council of Economic Advisers, 2016). After the near meltdown of the financial system and the economic fallout of the coronavirus pandemic, attitudes towards active labor market and social policies might have changed. Therefore, MMT may provide a welcome academic justification for these policies. Moreover, the popularization of MMT through the blogosphere may have a profound effect on U.S. politics and economic policy in the 2020s and 2030s (Brady 2020).

A special feature of MMT and its policy recommendations is public debt. According to MMT, public expenditures can be financed by public debt or even by printing more money without negative economic side effects such as inflation, crowding-out of investments or national insolvency (Forstater 1999; Mosler 1998). The only precondition is that the respective state has its own currency. This is the most provocative conclusion of MMT proponents.

MMT is not a new theory that emerged from the financial crisis of 2008. Most of the policy recommendations can be found in the work of Lerner (1943, 1944, 1951), dubbed functional finance, as also mentioned by MMT proponents. The theory itself is Post-Keynesian and monetary. Post-Keynesian economics (Arestis 1996; Lavoie 2009) is the general heading for very different economic concepts and theories that rely on Keynesian economics, but that do not accept New Keynesian concepts (Dixon and Rankin, 1995). Meanwhile, economists of this tradition formed a group whose common feature is a so-called coherent financial stock-flow accounting framework (Godley and Lavoie, 2012, p. 12, who also sketch the development of MMT; Nikiforos and Zezza, 2017). As will become clear in the following, *ex post* accounting identities play a crucial role in MMT.

## Literature Review

Although there are a number of recent assessments of MMT, these contributions either do not contain a formal analysis (Brady 2020; Coats 2019; Epstein 2020; Hartley 2020; Newman 2020; Palley 2015a; Skousen 2020) or the formal analysis is a bit too sophisticated to isolate exactly where the theoretical foundation of MMT fails (Palley 2015b). Palley (2015a) discussed the elements of MMT with Tymoigne and Wray (2013) concluding that what MMT adds to old Keynesian economics is wrong. Similarly, Skousen (2020) investigated the macroeconomics textbook on MMT by Mitchell et al. (2019) concluding that MMT is dangerous as its policies may provoke runaway inflation, and that it is not required as countries can reduce unemployment substantially without applying MMT policies. Brady (2020) summarized five cornerstones of MMT concerning the sustainability of very high public debt and refuted them with results from old and contemporary economic literature. Coats (2019) studied MMTs free-borrowing hypothesis for governments and argued that this radical view was based on the critical assumption that the natural rate of interest is zero. Hartley (2020) found that MMT might be a political movement rather than an economic theory, as long as there is no empirical evidence for its propositions on government debt and inflation-free money creation. The MMT critique of Epstein (2019, 2020) is related to the existing institutions that are responsible for monetary and fiscal policy. According to Epstein, this institutional setting and the functioning of modern financial markets may seriously limit the implementation of MMT’s policy recommendations. Kashama (2020) assessed MMT from the viewpoint of macroeconomic stabilization in the eurozone. His conclusion was that the policy assignment to the governments and the central bank, with the central bank responsible for price-level stability, should not be changed, in stark contrast to MMT. Compared to these papers, this short contribution relates to the theoretical foundation of MMT at a very fundamental level.

This paper most closely resembles Palley (2015b). Palley provided a sophisticated theoretical analysis of MMT from a Keynesian viewpoint. He demonstrated very clearly the basic Keynesian approach of MMT and argued that nothing of relevance was added that would justify the term MMT. In contrast to Palley (2015b), this paper takes MMT seriously in the sense that a simple version of MMT is used to prove that it is identical to the Keynesian cross model. In the model, MMT’s approach of financing government expenditures by money creation is applied, showing that MMT’s interpretation of money does not change anything. MMT does not present a new theory of money, but only accounting identities. Moreover, the fundamental flaw in MMT is a misreading of the equilibrium condition of the underlying macroeconomic system. Far reaching policy recommendations, such as financing large-scale social policy expenditures by public deficits or printing money, do not seem to be justified on the basis of MMT. Moreover, information in the Online Supplemental Appendix shows that even in MMT, *ex post* Ricardian equivalence must hold true. This implies that money is neutral in the sense that it does not eliminate or mitigate the fiscal burden of government expenditures.

## Simplest MMT Model: SIM

The following presentation of MMT in the simplest version (SIM) is based on Godley and Lavoie (2012, pp. 61–72). SIM is interpreted as the basic model of MMT. Moreover, all subsequent extensions of the model inherit the characteristics of SIM. The notation in this presentation is somewhat modified (without any content change) to make it easier to compare SIM with the simplest Keynesian model in the next section. The disposable income of households, ${Y}_{d}$, is given by:

1 $${Y}_{d}=W\cdot {L}_{S}-T,$$

where *W* is wage, ${L}_{S}$ is labor supply, and *T* is tax payments of households. Note that firms are not modelled explicitly, as is quite usual in very simple macroeconomic models. Implicitly, firms employ labor services of households to produce goods and services and they pay wages to the households as remuneration of labor services.

SIM has two behavioral equations. The first one is the tax function, *T*, defined by the government:

2 $$T=t\cdot W\cdot {L}_{S}, t<1,$$

where *t* is the tax rate of a proportional wage tax. The second behavioral equation is the consumption function, *C*, of households:

3 $$C\left({Y}_{d},{M}_{HH-1}\right)=\alpha \cdot {Y}_{d}+\beta \cdot {M}_{HH-1}, 0<\beta <\alpha <1,$$

where $\alpha ,\beta$ are coefficients and ${M}_{HH-1}$ is money stock of households from the previous period. The consumption function in Eq. (3) depends on the disposable income, with α as the marginal propensity to consume and *β* as the influence of the money stock households hold from previous periods.

Money is created by the government via the public budget deficit:

4 $$\Delta {M}_{G}={M}_{G}-{M}_{G-1}=G-T,$$

where ${M}_{G}({M}_{G-1})$ is money creation of the government in the current (previous) period and *G* is government expenditures for goods and services. Equation (4) can be understood as the monetization of debt (Protopapadakis and Siegel, 1986; Thornton 2010). Instead of I-owe-you’s (IOUs), the government buys goods and services by creating its own money, also called outside money (Wray 2014). Money is defined here as an accounting measure, or “as a two-sided balance sheet phenomenon” (Bell 2001, p. 151). Therefore, it cannot be said whether it is an asset or only a numeraire (for a discussion of the latter, see Otaki 2012).

Households adjust their holding of money as follows:

5 $$\Delta {M}_{HH}={M}_{HH}-{M}_{HH-1}={Y}_{d}-C\left(=S\right),$$

i.e., the difference between disposable income and consumption is equal to the change in money holding. Obviously, the difference between disposable income and consumption must be equal to households’ savings, *S* (note that *S* is not included in SIM). National income is given by the production of consumption goods and public goods:

6 $$Y=C+G.$$

Note that Eq. (6) is an *ex post* identity. Therefore, it is neither right nor wrong. In addition, there are no investments. The proceeds are distributed to the factor of production, i.e., the labor services of households: $Y=W\cdot {L}_{D} \underset{}{\Rightarrow } {L}_{D}=\frac{Y}{W}$, where ${L}_{D}$ is labor services demand.

Since the money created by the government (money supply) must be equal to the money holding of households (money demand), the public budget deficit is equal to the change in the stock of money and, hence, savings:

7 $$\Delta {M}_{G}=\Delta {M}_{HH}\underset{}{\Rightarrow } G-T={Y}_{d}-C\left(=S\right).$$

Put differently, this means (not contained in the SIM presentation of Godley and Lavoie, 2012):

8 $$S=G-T.$$

Equation (8) is the implication of a standard economic circular flow model with government, where $S=I+(G-T)$, if there are no investments (as is the case in SIM), i.e., $I=0$. Obviously, the equality of savings, money creation and public budget deficit is a consequence of the descriptive circular flow model of the economy. This demonstrates that no new theory of money is presented with SIM and, hence, MMT. Instead, Eqs. (6, 7, 8) are *ex post* identities.

In a (long-run) steady state equilibrium, government expenditures must be tax financed in order to avoid so-called Ponzi-games:

9 $$G=T=t\cdot W\cdot {L}^{*}=t\cdot {Y}^{*},$$

with Y* as the steady state equilibrium national income. Rearranging the terms in Eq. (9) yields:

10 $${Y}^{*}=\frac{G}{t}.$$

Equation (10) is called fiscal stance. Godley and Lavoie (2012, p. 72) emphasized the importance of the fiscal stance as follows: “It [i.e., *G*/*t*] plays a fundamental role in all of our models with a government sector, since it determines GDP (i.e., gross domestic product) in the steady state.” In MMT, the expression *G*/*t* (government expenditures divided by the tax rate) is considered causal for the equilibrium national income, Y*. Even in a larger model with government money and portfolio choice (Godley and Lavoie, 2012, p. 99), the steady state solution collapses to Eq. (10) if the average interest rate on all government liabilities is zero (Godley and Lavoie, 2012, p. 115). A further implication (not mentioned) of SIM is again an *ex post* identity:

11 $$G-T=0 \underset{}{\Rightarrow }{Y}_{d}-C=0\underset{}{\Rightarrow }S=0.$$

This implication is consistent with the circular flow model of the economy since there are no investments in SIM: $I=0 \underset{}{\Rightarrow }S=G-T, G=T\underset{}{\Rightarrow }S=0$.

To summarize, the simplest model containing the main elements of MMT is based on the descriptive circular flow model of an economy, combined with a tax function defined by the government, and a consumption function. However, the conclusion suggests that government expenditures (in combination with the income tax rate) causally determine the equilibrium national income.^1^ To understand SIM better, it is compared with the simplest Keynesian model (KEYSIM) in the following.

## SIM Versus the Keynesian Cross, KEYSIM

The Keynesian cross model, or KEYSIM, can be considered the simplest Keynesian model of an economy. It can be found in any introductory macroeconomics textbook (Beck and Prinz, 2018, p. 145–156). The KEYSIM is also based on Eq. (6), i.e., that national income can be used for private consumption, *C*, or public expenditures for goods and services, $G\; (Y=C+G)$:

Moreover, the consumption function is given by:

12 $${C=C}_{0}+\alpha {Y}_{d},$$

i.e., consumption consists of an income-independent element, *C*_*0*_, and depends on disposable income, *Y*_*d*_, with α as the marginal propensity to consume. Disposable income is given by total income, *Y*, minus savings, *S*, and tax payments, *T*: ${Y}_{d}=Y-S-T$, whereby the tax is again a proportional income tax:

13 $$T=t\cdot Y.$$

Furthermore, in equilibrium, all government expenditures are financed via taxation so that $G=T$. Finally, since there are no investments, the circular flow model implies that savings are zero ($S=0$). Therefore, combining Eqs. (6, 12, 13) gives:

14 $$Y=C+G={C}_{0}+\alpha \left(Y-T\right)+T={C}_{0}+\alpha \left(Y-tY\right)+tY.$$

Solving Eq. (14) for the equilibrium national income, *Y*, yields:

15 $$Y-\alpha Y\left(1-t\right)-tY={C}_{0}, \underset{}{\Rightarrow } {Y}^{*}=\frac{{C}_{0}}{(1-\alpha )(1-t)}.$$

Equation (15) deviates from Eq. (9) ($G=T=t\cdot {Y}^{*}=t\cdot W\cdot {L}^{*}$) that also determines the equilibrium value of government expenditures. According to Eq. (15), the value of government consumption is given by:$G=t\cdot {Y}^{*}=t\cdot \frac{{C}_{0}}{(1-\alpha )(1-t)}$. In SIM, Eq. (3) says $C\left({Y}_{d},{M}_{HH-1}\right)=\alpha \cdot {Y}_{d}+\beta \cdot {M}_{HH-1}$. For sake of simplicity, let

16 $$\beta \cdot {M}_{HH-1}=A={C}_{0},$$

which is that part of consumption that is independent of current income. Note that the term $\beta \cdot {M}_{HH-1}$ in the consumption function is the only innovation in SIM, in comparison to KEYSIM. Accordingly, Eq. (14) holds also in SIM:

17 $$Y=C+G=A+\alpha \left(Y-T\right)+T={C}_{0}+\alpha \left(Y-tY\right)+tY.$$

The long-run steady state equilibrium national income with a balanced public budget reads according to Eq. (15). There is also no contradiction to the long-run steady state equilibrium of SIM in Eq. (10) (${Y}^{*}=\frac{G}{t}$) since this also implies in SIM:

18 $$G=T=t\cdot {Y}^{*}=t\frac{{C}_{0}}{\left(1-\alpha \right)\left(1-t\right)},$$

which is identical to the value of government consumption in KEYSIM, as can be seen by multiplying Eq. (15) with the tax rate, *t*.

Hence, up to this point, SIM and KEYSIM are indistinguishable. However, the Keynesian cross is an oversimplification of the Keynesian model. In this paper, only the short run is considered. Extending the model requires the incorporation of price-wage adjustments with Philips-curves. In such an extended model, price-wage dynamics will lead back to the long-term equilibrium. In contrast, MMT models do not contain price-wage adjustments. It is unclear what role money would play in MMT concerning price-wage adjustments. In this respect, MMT cannot be compared with a Keynesian model as applied here.

In addition, even in a neoclassical world with fully flexible wages and prices, the equilibrium condition (that may be written as ${Y}^{*}=Y$) will hold. Nevertheless, in neoclassical theory, supply determines equilibrium output. Moreover, with fully flexible prices and wages, monetary policy determines nominal variables in equilibrium. Fiscal policy may change the composition of demand and the distribution of income as fiscal stabilization is not an issue. Hence, in effect, the above analysis is not only compatible with MMT and Keynesian theory, but also with neoclassical macroeconomic theory. Consequently, SIM (and MMT) is not wrong. Where then does MMT get it wrong?

## Misreading the Equilibrium Condition

The key to understand MMT is reading the equilibrium result in Eq. (18). By simple algebra, this equation can be written as:

19 $${Y}^{*}=\frac{G}{t}=\frac{{C}_{0}}{(1-\alpha )(1-t)}=\frac{{\beta M}_{HH-1}}{(1-\alpha )(1-t)}.$$

As an equation, it can be interpreted in several ways:

1. National income, Y*, is determined by the government via choosing expenditures, *G*, and tax rate, *t*.
2. National income, Y*, is determined by the income-independent part of consumption, ${C}_{0}=\beta {M}_{HH-1}$, the marginal propensity to consume, *α*, and the tax rate, *t*.
3. National income, Y*, is the result of the aggregate demand in an economy.
4. The production side of national income, Y*, determines private and public consumption.
5. Aggregate production and aggregate demand are equal at the equilibrium national income of Y*.

All of these versions are of necessity correct, or at least not wrong, because there is no causality involved. Since both models share the same bases (i.e., the circular flow model of an economy, a tax function and a consumption function) and the same equilibrium condition (aggregate supply is equal to aggregate demand), they are indistinguishable. Moreover, it is clear that both models are of Keynesian origin because the supply side reacts passively to changes in aggregate demand. By assumption, aggregate demand determines (is causal for) national income.

The claim of MMT that government expenditures, financed by running a public deficit via the creation of money, determine (causally) national income constitutes a misreading of an equilibrium condition (i.e., reading the equation from right to left). However, an equation simply equates two sides of the equation and nothing else. The causality is externally added by the reader, as it were.

**Fig. 1:**
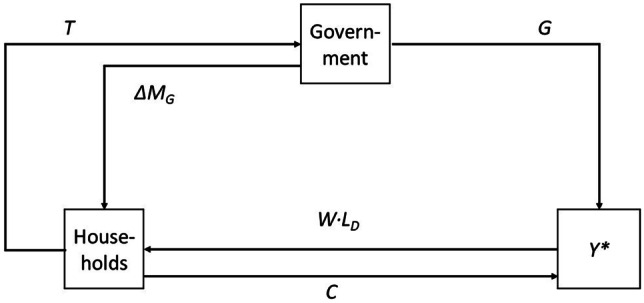
Fiscal stance and national income determination. Source: Own depiction

Figure 1 shows SIM in a circular flow diagram. According to MMT, government expenditures for goods and services, *G*, in combination with a public budget deficit financed by creation of additional money, Δ*MG* (i.e., that part of *G* not financed via taxation with the tax rate, *t*), determines national income, Y*.

However, as Fig. 1 demonstrates, all causal explanations of Y* are circular. The model contains not one, but two decision making units: the government and households. Therefore, both are causal (in an interdependent way) for the size of national income. Moreover, the model is built on *ex post* identities (i.e., on accounting identities) as Fig. 1 demonstrates.

Another proposition of MMT can be clarified with Fig. 1. According to MMT, it is neither taxes nor borrowing that finance public expenditures, but the creation of fiat money (Forstater 1999, citing Lerner 1951; Bell 2000). In Fig. 1, this corresponds with $G=\Delta {M}_{G}$. This implies that *T* = 0. However, even in SIM, the steady state equilibrium requires that the No-Ponzi-Game condition, *G* = *T*, holds true (Godley and Lavoie, 2012, p. 71). In Fig. 1, the circular flow between households and the government indicates the equivalence of taxes and fiat government money according to $T=\Delta {M}_{G}$. Insofar, taxation is a method to regulate the amount of money in the economic circuit (Tymoigne and Wray, 2013). However, this is only an *ex post* accounting identity. As indicated by Fritz Machlup, *ex post* identities are futile for policy conclusions:

*“Macro-theorists have not always been careful and have repeatedly been misled into thinking they could deduce consequences from an ex post definition, for example, that they could deduce the effects of an increase in investment from the definitional equation Y = C + I. This is logically impossible, and therefore inadmissible in macro-theory and in micro-theory”* (Machlup, 1963, p. 120).

Furthermore, the misreading of the equilibrium condition of SIM is responsible for the policy recommendations. Equation (12) and all equations containing Y* are different versions of the same equilibrium condition (e.g., Eq. (15)). Of course, static multipliers may be derived from Eq. (14). Since the basis of MMT is the old-school Keynesian cross model, changes in aggregate demand variables lead to certain static multipliers. In effect, that government expenditures may increase national income does not depend on a certain theory of money, but on the fact that such a model allows by assumption only demand-side effects. That is all one can say on fiscal policies in this model.

Figure 1 also sheds some light on the issue of inflation, which is not a problem according to MMT. Inflation only occurs when aggregate demand is larger than aggregate supply. If demand outstrips supply, the government can decrease money supply by increasing taxes. Figure 1 shows that there is no monetary theory in this model, no assumptions about the endogeneity of money supply, the role of excess reserves of the central bank, the role of the financial sector and people’s expectations concerning the effects of monetary policy. If, for example, people expect more inflation or taxes as a result of higher government debt, the simple results of the SIM may not hold.

Figure 1 also shows another flaw of MMT. It neglects the role of the foreign sector. MMT assumes that as long as a country does not borrow in a foreign currency, it cannot default. This is certainly true, but most countries do not have the exorbitant privilege (Eichengreen 2011) of issuing a reserve currency. They have no choice but to borrow in foreign currencies. This aspect of MMT may explain why MMT is more popular in the U.S. than in other countries. The propositions of MMT may cause serious financial instability in an open economy with flexible exchange rates as fixed exchange rates would impose a hard budget restraint on the government which would mean that the government could default on its debt.

## MMT and Economic Policy

At first glance, it seems that MMT and the almost worldwide monetary policy called quantitative easing (QE) have much in common. In MMT as with QE, the central bank creates very large quantities of money, mainly by buying government securities in the secondary market. The similarity ends there. QE is designed as a temporary policy in order to stabilize economies which suffer from financial crises, such as that caused by a pandemic virus. QE is not and was never intended to finance government expenditures (Globerman 2020). Central banks will start to reduce the quantity of money after the crises by selling back government securities before they mature (Globerman 2020). Although QE means a certain degree of monetizing government debt, it remains a policy instrument of a politically independent central bank (Epstein 2019).

In contrast, in MMT the government finances public expenditures via money creation, with no intention to refinance them with taxes (Bell 2000). That is, monetization of the debt is forever. In this way, politicians control the creation of money and not politically independent central banks. Moreover, monetary policy explicitly finances government expenditures. Monetary policy is no longer monetary policy, but rather a combination of monetary and fiscal policy (Tymoigne 2016). As is recognized by serious proponents of MMT (Mitchell 2010a, 2010b), such a policy can only last as long as there are spare capacities in an economy in the form of unemployed workers and underused production facilities. If capacity is fully used, additional money will create inflation. At this point, the government should increase taxes to avoid inflation by restricting private resource use via consumption and investment. In contrast to QE, the creation of money (or, equivalently, the monetization of government debt) in MMT is an instrument to finance public expenditures. Taxes serve as instruments to reduce private consumption and investment, in order to avoid inflation. However, there are also new ideas to employ taxes for financing social policy and even a Green New Deal (Baker and Murphy, 2020).

The differences between QE and MMT demonstrate that MMT has different political intentions. Monetary policy is employed to finance the state in order to release taxation from its usual function of financing public goods. Another policy recommendation underlines this intention, the so-called job guarantee (JG) (Mosler 1998; Parguez 2008; Tcherneva 2020). JG “is at the centerpiece of MMT reasoning. It is neither an emergency policy nor a substitute for private employment, but would become a permanent complement to private sector employment” (Mitchell et al., 2019, p. 295). JG is considered as an automatic stabilizer in MMT (Mitchell et al., 2019, p. 303) and would be financed by money creation, i.e., public debt. Although it has some resemblance to Keynesian deficit-financed stabilization policies in a recession, guaranteeing jobs that produce goods and services at the minimum wage is outside the Keynesian concept. In effect, it is labor market policies paid for by money creation. However, that JG policy may become inflationary is denied (Mitchell et al., 2019, p. 304) because the government is “buying labour off the bottom” (Mitchell et al., 2019, p. 304), i.e., that minimum-wage JG-employment has no effect on the structure of wages. Moreover, MMT ignores all microeconomic problems of JG policy.

This brief look at the differences between QE and MMT demonstrates that the monetary concept of MMT has almost nothing in common with QE. The MMT policy intentions promise a kind of new brave world that is economically stable, socially more equal and environmentally green. However, the economics of MMT are unclear at best. Who will pay for this world remains an unanswered question. As MMT seems to suggest, it is a free lunch.

As a matter of fact, someone has to pay sometime for the economic, social and environmental benefits of MMT. Since taxes are excluded and public deficits are monetized, the inflation tax is financially the last resort, unless it is avoided by taxes. Hence, the usual result is still valid. The usage of real resources must be paid for, either through ordinary taxes, the inflation tax or financial repression.

This leads to the final point of the analysis as MMT neglects the political aspects of recommended policies. MMT hands over responsibility for fiscal and monetary policy to politicians seeking re-election, hoping that these politicians will act responsibly. Therefore, MMTs over-simplistic analysis understates the risks of the policy implications (Palley 2015b). For policy recommendations, larger sets of behavior functions are required that show how households and firms react and adjust to such policies (Machlup 1963). Mankiw (1988), Reinhorn (1998) and Otaki (2007) incorporate imperfect competition into the Keynesian cross model. Therefore, one can say that the policy implications and recommendations of MMT are neither theoretically well-founded nor politically justified (for further critical reviews of MMT, see e.g. Brady 2020; Newman 2020; Skousen 2020).

## Conclusion

The main contribution of this paper to the literature on MMT concerns the theoretical foundation of MMT. In a simple macroeconomic model, SIM, it is shown that MMT is indistinguishable from the Keynesian cross model, as well as neoclassical macroeconomic models. Demonstrating this with models is a necessary step to demystifying and debunking MMT as an economic theory.

There are few cases where many economists, Keynesian or Austrian, agree, but the rejection of MMT’s hypotheses is one of them (Brady 2020; Skousen 2020). In the current paper, simple macroeconomic models were applied to show that there is almost nothing new in MMT. The important insight is that the fundamental role of the so-called fiscal stance in MMT (i.e., equilibrium national income is equal to government expenditures divided by the tax rate on income, ${Y}^{*}=\frac{G}{t}$) is a relationship that holds trivially true in all Keynesian cross models and even in neoclassical macroeconomic models. It is neither specific to MMT nor does it follow from a new theory of money.

In fact, the fiscal stance is the consequence of the *ex post* identities of the economic circuit, an aggregate consumption function of private households and the non-Ponzi-game condition for the state. In the SIM model, the latter condition renders money meaningless because it is by definition an accounting identity, and because output used by the state can no longer be consumed (or saved) by private households. Ultimately, government expenditures are financed by taxes, whatever they are called. Moreover, it is not possible to say that the government can determine equilibrium national income. This statement is a misunderstanding of the fiscal stance that is an equilibrium condition, without any causality whatsoever.

Furthermore, MMT does not provide a theory of money. Instead, “money is a creation of the state” is the simple statement on which money is based (which is the topic of Knapp’s “The State Theory of Money”, published in German in 1905; MMT theorists quote this origin). However, in comparison to the conventional theory of money, this is a big step backwards. Last but not least, the far-reaching policy recommendations of MMT are not justified by economic theory. They are highly exaggerated since no further behavioral assumptions for households or firms are formulated that could show how the respective economic entities react and adjust to the recommended policies.

## Supplementary Information

Below is the link to the electronic supplementary material.

Supplementary file1 (DOCX 20 KB)

## Footnotes

1 The Online Supplemental Appendix shows in a two-period variant of SIM that in MMT *ex post* Ricardian equivalence must hold true. The reason is that government expenditures use real economic resources that must be transferred from private households to the state. The instrument to carry out this transfer is called taxes.
