# Supplementary material for: Modern Monetary Theory: A Solid Theoretical Foundation of Economic Policy?
Source: Atl Econ J. 2021 May 25;49(2):173–86. doi: 10.1007/s11293-021-09713-6 (PMC8144874; doi:10.1007/s11293-021-09713-6)
Supplement: Supplementary file 1 — Supplementary file1 (DOCX 20 KB) [file 11293_2021_9713_MOESM1_ESM.docx]

**Online Supplemental Appendix**

**to**

**“Modern Monetary Theory: A Solid Theoretical Foundation of Economic Policy?**

This appendix shows that *ex post* Ricardian equivalence is consistent even with MMT.

A two-period macro-model is considered:

$Y_{1}=C_{1}+G_{1}$, and (A1)

$Y_{2}=C_{2}+G_{2}$. (A2)

Suppose that public expenditures in the first period are financed by fiat money, i.e.,

$G_{1}=M_{G}=M_{HH}, T_{1}=0$. (A3)

The consumption function is given by Equation (3) in the main text:

$C\left( Y_{d},M_{HH-1} \right)=\alpha\cdot Y_{d}+\beta\cdot M_{HH-1}, 0<\beta<\alpha<1$.

Note, however, that there is no previous period to period 1; hence: $M_{HH-1}=0$ and

$C_{1}=\alpha\cdot Y_{d1}, Y_{d1}=Y_{1}-T_{1}-S; T_{1}=0\overset{\Rightarrow}{}Y_{d1}=Y_{1}-S\overset{\Rightarrow}{}C_{1}=\alpha(Y_{1}-S)$. (A4)

Combining Equations (4) and (5), i.e., the supply of money is equal to the demand of money, in the main text,

$\Delta M_{G}=M_{G}-M_{G-1}=G-T$ , and (4)

$\Delta M_{HH}=M_{HH}-M_{HH-1}=Y_{d}-C(=S)$ , (5)

yield:

$\Delta M_{G}=M_{G}-M_{G-1}=\Delta M_{HH}=M_{HH}-M_{HH-1}=Y_{d}-C=S$. (A5)

In the first period $M_{G-1}=M_{HH-1}=0$, this implies:

$M_{HH}=S$. (A6)

The determination of the equilibrium national income in the first period gives:

$Y_{1}=\alpha\left( Y_{1}-S \right)+G_{1}=\alpha\left( Y_{1}-M_{HH} \right)+M_{HH}$,

$\overset{\Rightarrow}{}Y_{1}^{*}=M_{HH}=S=G$. (A7)

This means that the national income of the first period will be completely absorbed by public expenditures that show up in the form of money in households.

In the second period, households are assumed not to save. Consumption in the second period is then given by:

$C_{2}=\alpha\cdot Y_{d2}+\beta\cdot M_{HH-1}=\alpha\left( Y_{2}-T_{2} \right)+\beta\cdot S$. (A8)

Moreover, public expenditures in the second period are tax financed:

${G_{2}=T}_{2}=t\cdot Y_{2}$. (A9)

Hence, equilibrium national income is given by:

$Y_{2}=\alpha\left( Y_{2}-t\cdot Y_{2} \right)+\beta\cdot S+t\cdot Y_{2}$. (A10)

Solving for *Y_2_* yields:

$Y_{2}^{*}=\frac{\beta\cdot S}{(1-\alpha)(1-t)}=\frac{\beta\cdot M_{HH-1}}{(1-\alpha)(1-t)}$. (A11)

To make this two-period model economically meaningful, the first period is interpreted as the birth of money period. Since the entire output is the money created, the first period has no other generic economic meaning. Moreover, since in the first period $Y_{1}^{*}=M_{G}=M_{HH}=G=S$, the amount of money created is completely arbitrary. This arbitrariness allows the equalization: $\beta\cdot M_{HH-1}=C_{0}$, because *C_0_* is also a completely arbitrary, exogenously determined variable.

In fact, this simple two-period model demonstrates that even in MMT *ex post* Ricardian equivalence holds true. The reason is very simple: Since the state uses real economic resources, they must be paid for with taxes whatsoever. Taxes are only the tool to transfer resources form private households to the state.
